# Early Identification of Preterm Neonates at Birth With a Tablet App for the Simplified Gestational Age Score (T-SGAS) When Ultrasound Gestational Age Dating Is Unavailable: Protocol for a Validation Study

**DOI:** 10.2196/11913

**Published:** 2019-03-12

**Authors:** Archana B Patel, Kunal Kurhe, Amber Prakash, Savita Bhargav, Suchita Parepalli, Elizabeth V Fogleman, Janet L Moore, Dennis D Wallace, Hemant Kulkarni, Patricia L Hibberd

**Affiliations:** 1 Lata Medical Research Foundation Nagpur, Maharashtra India; 2 RTI International Research Triangle Park, NC United States; 3 M&H Research, LLC San Antonio, TX United States; 4 Department of Global Health Boston University School of Public Health Boston, MA United States

**Keywords:** gestational age assessment, last menstrual period, mHealth, newborn, prematurity, ultrasound

## Abstract

**Background:**

Although rates of preterm birth continue to increase globally, identification of preterm from low birth weight infants remains a challenge. The burden of low birth weight vs preterm is greatest in resource-limited settings, where gestational age (GA) prior to delivery is frequently not known because ultrasound in early pregnancy is not available and estimates of the date of the mother’s last menstrual period (LMP) may not be reliable. An alternative option is to assess GA at birth to optimize referral and care of preterm newborns. We previously developed and pilot-tested a system to measure the simplified gestational age score (SGAS) based on 4 easily observable neonatal characteristics.

**Objective:**

The objective of this study is to adapt the scoring system as a tablet app (potentially scalable approach) to assess feasibility of use and to validate whether the scoring system accurately predicts prematurity by itself, over and above birth weight in a large sample of newborns.

**Methods:**

The study is based in Nagpur, India, at the Research Unit of the National Institute of Child Health and Human Development’s Global Network for Women’s and Children’s Health Research. The Android tablet app for the SGAS (T-SGAS) displays de-identified photographs of skin, breasts, and genitalia across a range of GAs and line drawings of infant posture. Each item is associated with a score. The user is trained to choose the photograph or line drawing that most closely matches the newborn being evaluated, and the app determines the neonate’s GA category (preterm or term) from the cumulative score. The validation study will be conducted in 3 second level care facilities (most deliveries in India occur in hospitals, and women known to be at risk of preterm birth are referred to second level care facilities). Within 24 hours of delivery, women and their babies who are stable will be enrolled in the study. Two auxiliary nurse midwives (ANMs) blinded to prior GA assessments will use the T-SGAS to estimate the GA status of the newborn. An independent data collector will abstract the GA from the ultrasound recorded in the hospital chart and record the date of the mother’s LMP. Eligibility for analysis is determined by the ultrasound and LMP data being collected within 1 week of each other to have a rigorous assessment of true GA.

**Results:**

Publication of the results of the study is anticipated in 2019.

**Conclusions:**

Until GA dating by ultrasound is universally available and easy to use in resource-limited settings, and where there are restrictions on ultrasound use due to their use for sex determination and abortion of female fetuses, this study will determine whether the T-SGAS app can accurately assess GA in risk categories at birth.

**Trial Registration:**

ClinicalTrials.gov NCT02408783; https://clinicaltrials.gov/ct2/show/NCT02408783 (Archived by Webcite at http://www.webcitation.org/75S2kmr3T)

**International Registered Report Identifier (IRRID):**

DERR1-10.2196/11913

## Introduction

An estimated 15 million children per year (11% of live births) are born prematurely (less than 37 weeks of gestation), predominantly in Africa and Asia [1]. Prematurity rates are increasing globally and of concern because prematurity is a well-recognized risk factor for increased neonatal mortality and morbidity [2-4]. In settings where the incidence and adverse consequences of prematurity are the highest (ie, resource-limited settings), pregnant women may not know the date of their last menstrual period (LMP) and ultrasound is often not available—particularly first-trimester ultrasound, when estimates of gestational age (GA) are more accurate [5]. Since premature birth may not be anticipated by health care providers prior to labor and delivery [6,7], it is important that prematurity be identified at birth so that preterm neonates receive effective interventions as soon as possible after delivery [8].

When prenatal GA is unavailable, it can be estimated at birth by physical examination of the newborn using scoring systems such as the 12-item New Ballard Score (NBS) [9] and the 22-item Dubowitz score (DWS) [10]. A simplified 11-item scoring system adapted from the NBS was developed by Meharban Singh in 1975 and is in use in India [11]. However, even the use of simplified GA scoring systems in first (Primary Health Centers) and second (district and regional healthcare centers) level facilities in resource-limited settings, where the majority of the world’s neonates are born, is difficult. Further, predicted GA from these scores has often been compared with women’s reports of their LMP, and is not a reliable gold standard, such as first trimester ultrasound [12]. In addition, these scoring systems have not been adequately evaluated for regional, geographical, racial or ethnic robustness. A pragmatic postnatal GA assessment tool that can be used by community birth attendants or auxiliary nurse midwives (ANMs) who deliver the majority of newborns in rural health settings is urgently needed to optimize referral and care of preterm neonates when prenatal GA is unavailable.

We previously used the NBS, DWS and Meharban Singh scoring system to develop a 4-item simplified gestational age score (SGAS) for use in low birth weight newborns in India [13]. The 4-item scoring system is as follows: posture (0 to 4), skin (–1 to 5), breasts (–1 to 4), and genitals (–1 to 4). The total score was used to define 4 SGAS categories: <32 weeks, ≥32 to <35 weeks, ≥35 to <37 weeks, and ≥37 weeks. The SGAS then underwent initial testing in 171 newborns [13] and was found to have a high positive predictive value for very preterm (<32 weeks gestation) and moderate preterm (32-35 weeks gestation) neonates compared with the NBS. In our prior study, a score of <14 predicted that a newborn was preterm (GA of <37 weeks). Agreement between independent raters for the SGAS was higher than that for the NBS (Cohen’s kappa 0.83 and 0.71, respectively) indicating that the SGAS was promising to assist with community-based triage and referral decisions for preterm neonates, but in need of further validation.

We elected to conduct a validation study after adapting the SGAS scoring system as a mobile health (mHealth) intervention for two reasons. First, the items used for the SGAS were derived from the NBS and the scoring is based on a description of the skin, breasts, and genitalia that require appropriate training when used by community health workers (such as ANMs) [14]. We proposed that it would be easier to train first level facility health care workers to estimate the score for each item if there was a photograph as well as a text description of the item. Second, since providing a standardized photograph for comparison would be easier using an mHealth app and, given the widespread availability and use of mobile phones, and even tablets, in resource-limited settings [15], this approach would be novel and potentially feasible. As recently reviewed by McBride et al [16], mHealth is increasingly being accepted by community health workers to improve knowledge about maternal and child health [17] and access to care [18], but there are few studies that have used mHealth apps to guide need for referral of neonates to second level care facilities. In this paper, we describe the development of a pictorial-based tablet app for the SGAS (T-SGAS) and its use as an assessment tool. The T-SGAS will be evaluated by ANMs, who are the frontline skilled birth attendants, particularly in first and second level care facilities in most of India, to assess GA compared with ultrasounds obtained as early as possible during pregnancy.

The Eunice Kennedy Shriver National Institute of Child Health and Human Development’s (NICHD’s) Global Network for Women’s and Children’s Health Research is a partnership and collaboration between 7 multidisciplinary research units and a Data Coordinating Center dedicated to improving maternal and child health outcomes and building health research capacity in resource-limited settings. Goals include testing feasible, cost-effective, sustainable interventions to provide guidance for the practice of evidence-based medicine. The research unit in Nagpur, India and the Data Coordinating Center at RTI International developed and will help validate the T-SGAS.

The objective of this paper is to describe the development of the T-SGAS, its preliminary assessment of feasibility, and a protocol to evaluate whether it accurately predicts prematurity in a large sample of newborns. The programmatic feasibility of using the app will be evaluated by assessing the agreement of GA assessments performed by 2 ANMs. The accuracy of the app will be assessed by comparing the assessment of preterm vs term-based newborns on LMP alone, ultrasound alone, and the combination of LMP and ultrasound.

## Methods

### Development of the Tablet App for the SGAS (T-SGAS)

The development of the T-SGAS was achieved through the following steps: (1) collection of a repository of photographs of the items in the SGAS for preterm and term newborns; (2) selection of photographs for the range of scores for skin, breasts, and genitalia and consensus of neonatology experts for the best photograph of each item score; and (3) development of the Android T-SGAS for visually matching the newborn to the photographs, which auto-calculates the total SGAS and assigns a GA after the user choses the appropriate photograph. A score of <14 is considered to represent preterm, as we had found in our prior study [13].

**Figure 1:**
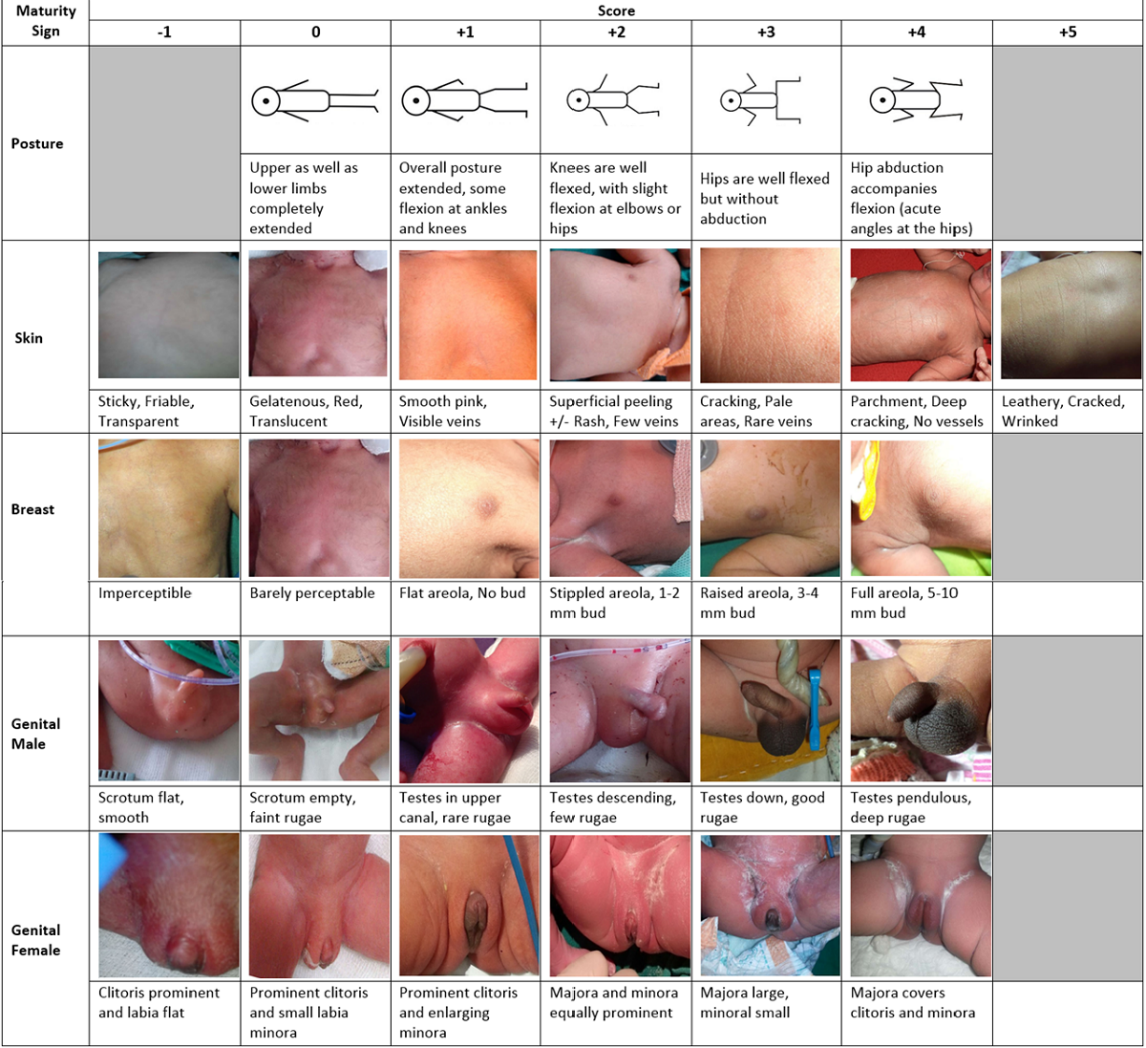
Photographs of the neonatal characteristics for the tablet app for the simplified gestational age scoring system (T-SGAS).

De-identified photographs of skin texture, breasts, and genitalia of 1800 newborns of varying GA within 24 hours of birth were obtained from five tertiary care hospitals in Nagpur, India, after obtaining written informed consent from the mother of the neonate. A panel of three senior neonatologists, trained in the use of the NBS, selected photographs that best matched the original text description of the development of the skin, breasts, and genitalia [9] based on agreement of at least two of the neonatologists. Since the goal was to select the most unambiguous pictures, whenever possible, we chose pictures selected by all three neonatologists to match each description. The neonatologists did not use the score to make their selection—the score was automatically assigned based on the text and picture description. The three neonatologists selected and matched the photographs together (not independently and not blinded to the interpretation of the other neonatologists). If more than one photograph matched an item and score, the photograph with the best clarity, lighting, and detail was selected. Posture was depicted by line diagrams instead of using photographs of newborns that could be identified. The 25 images selected by the neonatologists (7 photographs of the skin, 6 of the breasts, 6 of the male genitalia, and 6 of the female genitalia) and the line diagrams of posture with their descriptions were embedded in the T-SGAS (see Figure 1). Note that the scoring range from –1 to +5 was based on the original Ballard Scoring System [9] and retained in the NBS System. For example, skin ranged from –1 to +5, breast and male and female genitalia ranged from –1 to +4, and pictures of posture ranged from 0 to +4. This is why there are 5 empty slots in Figure 1. Each item has an associated score. The T-SGAS then auto-calculated the total score and classified the newborn’s GA. The conversion of T-SGAS to GA categories is shown in Table 1.

The T-SGAS was designed to collect data in 5 different forms using the same tablet (forms SGAS01-SGAS05). Forms SGAS01 and SGAS02 were used by ANM-1 and ANM-2 to assess GA, blinded to each other’s assessment. Form SGAS03 contained data on demographic details of the mother and the newborn, foot length of the newborn, GA based on LMP, and GA based on the earliest ultrasound in pregnancy to determine study eligibility. Form SGAS04 was a random sample verification form for quality assurance by the master trainers to assess the GA, as well as to verify the details from the source documents. Form SGAS05 recorded any protocol deviations or unexpected events in the course of the neonate’s enrollment.

The T-SGAS was written in Java and was developed using the Eclipse platform to run on Android-based tablets, as a cost-effective and easy-to-use app that includes support for multiple-languages, enables field data collection in low- and middle-income countries, and has capabilities to integrate diverse data management methodologies. We would also consider developing a version of the T-SGAS for iOS should there be interest from potential users. The software was developed as a suite of software tools for form design, implementation, and reporting. Various data management models were developed to work in diverse conditions including: SIM based tablet or Wi-Fi transmission with Dropbox/Dropsync integration, File Transfer Protocol (FTP), migration of data over SD cards or USB cables and use of Wi-Fi capable external drives. Confidentiality of the data was ensured through encryption of the data on the tablet and during upload to Dropbox or the FTP site. Access to the data are limited to a few personnel who work on the server, which is behind a firewall. The T-SGAS was tested frequently during development for usability and reliability on SIM card–enabled Android tablets. The final T-SGAS was secured by user-specific passwords.

Built-in validations and range checks were implemented as needed on questions to prevent users from moving to the next question on the form if the validation failed. Examples of system-checks and automation include:

- Based on the eligibility questions on the first data collection form (SGAS01), the T-SGAS automatically generated the rest of the forms for potentially eligible subjects (although the potential accuracy of GA was not known at this stage).
- Based on gender (male or female) the app presented the 3 neonatal characteristics with the correct gender assignment as the fourth neonatal characteristic.
- Cross form checks were also programmed for questions (such as the current status of the baby, sex of the baby, etc).
- To reduce errors, key fields from the enrollment form SGAS01 (such as date and time of delivery, consent obtained, etc), were also programmed to be present on the introduction screens of forms SGAS02 to SGAS05, which recorded the results of the actual GA assessment.

**Table 1:** Conversion of the tablet app for the simplified gestational age scoring system (T-SGAS) to gestational age categories [13].

T-SGAS total score	Gestational age (weeks)
<7	<32
7-9	≥32 to <35
10-13	≥35 to <37
>14	≥37

### Study Design for the T-SGAS Validation Study

The study is a multicenter evaluation of the T-SGAS that will be conducted in three second level care birthing facilities in and around Nagpur, India (Daga Memorial Hospital, Nagpur; Government Hospital, Bhandara; and Government Hospital, Wardha), where most of the deliveries are conducted by ANMs. Each facility will have a team of ANMs and data collectors trained to use the T-SGAS. Over the study period, two ANMs will be available at all times to independently use the T-SGAS to calculate the newborn’s GA within 24 hours of birth. Since the ANMs will be blinded to the neonates actual GA, the data collector will obtain the actual GA data by assessing the mother and her chart, to ascertain her neonate’s eligibility for analysis. This will be done after the ANMs have assessed all neonates whose mother has consented to her newborn participating in the study.

### Eligibility Criteria for the Newborns

During 12-hour study shifts, all mothers who meet the inclusion criteria and have no exclusion criteria (Textbox 1) will be informed about the study and invited to participate. Exclusion criteria were determined on the basis of conditions that could interfere with the assessment of the neonate’s posture. Mothers who are willing to participate will complete a consent process and sign the consent form.

Since it is frequently not possible to obtain and confirm the estimated GA by ultrasound, we decided *a priori* to have additional exclusion criteria for analysis eligibility, to improve the likely validity of the GA estimated by ultrasound, as follows:

- Women whose estimated GA from the date of the LMP is not within 1 week of the GA estimated from the ultrasound;
- Neonates with GA at birth <20 weeks or >44 weeks based on either the date of the LMP or ultrasound;
- Two independent assessments of the newborn were not available within the first 24 hours of life; and
- ANMs who had not assessed at least 100 newborns during the study were unavailable.

### Ethics and Consenting

The protocol and the informed consent documents were submitted and approved by the Institutional Review Boards (IRBs) and ethics committees of the Lata Medical Research Foundation IRB (FWA00012971), the Partners Human Research Committee, Boston, MA, and the Boston University Medical Campus IRB. This study is supported by The Eunice Kennedy Shriver NICHD’s Global Network for Women’s and Children’s Health Research. The study was registered on ClinicalTrials.gov (NCT02408783).

### Study Variables

Study variables include the date and time of the neonate’s delivery, delivery outcome, gender, birth weight, and maternal demographic data, including level of maternal education (none, primary, secondary, postsecondary), mode of delivery (vaginal, cesarean section, vaginal assisted), birth weight (grams), date of her LMP, and GA per the hospital-based ultrasound report.

### Implementation of the Study Protocol

Three senior neonatologists with experience in GA assessment at birth and an obstetrician (master trainers) will train the ANMs on the use of the T-SGAS. The training will consist of 4 days of classroom teaching sessions, which include training on Good Clinical Practice guidelines, how to examine the newborn, the different aspects of the 4 physical characteristics of the newborn at different GA, and how to use the T-SGAS. The ANMs will be trained in 3 batches and a final one-day re-orientation training for all ANMs will be conducted prior to the start of the study. The ANMs will then participate in a two-week practical training in the study hospitals. The first week of practical training will be conducted in the delivery room, postnatal wards, and in the special care neonatal units. Training includes adherence to asepsis protocols of the nursery, as well as one-on-one training of how to use the T-SGAS for assessing singleton live births, conducted by the master trainers. The second week, the trainees will independently assess 30 newborns of varying GA before these babies are independently reassessed by the master trainer. ANMs whose scores for each item agree with the master trainer’s score at least 80% of the time will be considered successfully trained.

**Figure 2:**
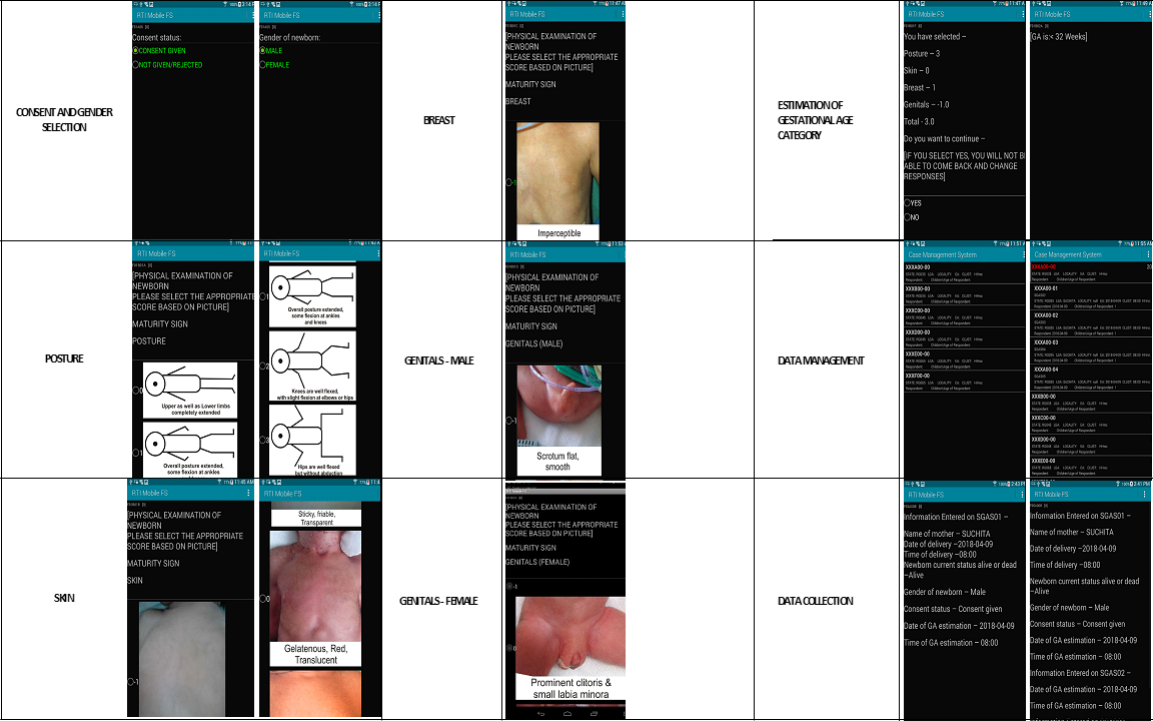
Tablet screens showing use of the tablet app for the simplified gestational age scoring system (T-SGAS).

The tablet-based forms are password protected and accessible only by using log-in credentials. After log-in, a Case Management Screen appears with the list of facilities and a preinstalled drop-down menu of identification numbers (IDs). The first ANM (ANM-1) sequentially allocates the IDs to the women who consented and delivered singleton live births. ANM-1 evaluates the newborn and uses her log-in credentials to open the electronic study form (SGAS01) that records date and time of delivery, gender of neonate, consent status, and then uses the touch screen to choose pictures that most accurately represent the neonate’s skin, breasts, genitals, and posture (Figure 2). The GA score is then auto-calculated and the neonate is classified into a specific GA group. On completion of the form, a pop-up warning message appears on the screen to verify the assessment and form entries. When verified, the form is saved and cannot be reopened by ANM-1. Completion of the form enables automatic population of subsequent forms with the same ID for the same participant. Color coding is used to identify the pending forms, so that ANM-2 can assess the same newborn using her log-in credentials within 24 hours of birth (using form SGAS02).

**Inclusion and exclusion criteria to participate in the tablet app for the simplified gestational age scoring system (T-SGAS) study.**

Inclusion criteria

- Mother had regular menstrual cycles prior to pregnancy
- Mother knew the date of her last menstrual period
- Mother has a report of at least one prenatal ultrasound assessment of gestational age during pregnancy (ideally in the first trimester)
- Mother and baby are clinically stable
- Mother delivered singleton neonate at the study hospital
- Neonate is within the first 24 hours of life

Exclusion criteria

- Neonates with birth asphyxia or who were resuscitated
- Neonates with major congenital anomalies or signs of neurological depression

**Figure 3:**
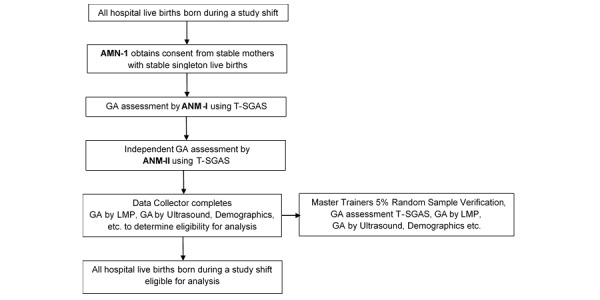
Overview of the T-SGAS study. ANM: auxiliary nurse midwife, GA: gestational age, T-SGAS: tablet app for the simplified gestational age scoring system, LMP: last menstrual period.

Data collectors are senior nurses trained to abstract study data from the hospital records. After ANM-1 and ANM-2 have completed their assessments, the data collector logs in to add the GA from the ultrasound report and the date of the LMP on form SGAS03. This procedure ensures that GA assessment by the T-SGAS is done by the ANMs who do not know the baby’s estimated GA at birth. Additional demographic details of the mother and the newborn, foot length of the newborn, and birth weight are included in this form. These data are used to determine final eligibility for analysis (Figure 3).

Periodic 2-day retraining will be provided to the ANMs and their assessments will have to meet the standard of 80% agreement of items with that of the master trainers. Focus group discussions for the master trainers and ANMs will be conducted to understand the limitations, challenges, and ease of use of the T-SGAS.

#### Quality Control Procedures

The master trainers will complete a GA assessment for 5% of the newborns selected at random to monitor accuracy of the ANMs’ assessment of GA. These data will be entered on form SGAS04. Since the ultrasounds are done for clinical purposes and may not be available at the hospital where the baby is delivered, there are no additional quality control procedures for estimation of GA from the ultrasound. If there is more than one ultrasound available, the earliest ultrasound in the pregnancy will be selected for the GA estimate, ideally one that has been conducted within the first trimester. The accuracy of the ultrasound will likely be improved by requiring the GA by ultrasound to be within 1 week of the mother’s assessment of GA based on her LMP. Form SGAS05 will be used to record any protocol deviations or unexpected events in the course of the neonate’s enrollment.

Data collection and transmission will be on a real time basis to an RTI International Server (Data Coordinating Center), which will allow daily viewing of data by data managers and statisticians. Edit reports (missing data, pending forms, inconsistencies between the forms and data) will be generated on a daily basis and sent to the birthing facilities so that the data collector can verify the data and resolve inconsistencies.

Monitoring will also be conducted by the research staff and master trainers during regular planned and unplanned visits to facilities to review ANM performance.

### Analysis

#### Sample Size

We have assumed that the prevalence of preterm births will be approximately 10% in this population. In a 2-sided test for sensitivity, a sample size of 7440 will achieve 80% power at a significance level of 0.05 when the sensitivity under the null hypothesis is 0.60 and the sensitivity under the alternative hypothesis is 0.65. This sample size will also be sufficient to achieve 80% power at a 0.05 significance level in a 2-sided test for specificity, when the specificity under the null hypothesis is 0.70 and the specificity under the alternative hypothesis is 0.75, which requires a minimum of 704 subjects.

#### Analysis plan

Firstly, we will use the Fleiss kappa statistic to estimate agreement between GA (as a dichotomous outcome) measured by ANM-1 and ANM-2. Since our goal is to evaluate the use of the T-SGAS by ANMs, we will not determine which AMN is “correct” (eg, by having a third AMN independently assess the GA of the baby to resolve disagreement). Instead, we will assess the screening accuracy (sensitivity, specificity, prevalence of preterm) of the T-SGAS for both ANMs using 4 different reference standards: LMP alone, ultrasound alone, either LMP or ultrasound, and both LMP and ultrasound (when GA by ultrasound is within 1 week of the GA estimated by the date of the LMP) [19]. We will also determine the predictive accuracy using the area under a receiver operator curve, based on predicted probability estimates obtained from logistic regression. We will determine from the data the optimal cutoff for the dichotomization of the T-SGAS using tree analysis. For this analysis, each of the four aforementioned reference standards will be separately considered as dependent variables and the birth weight and the T-SGAS as independent variables.

Secondly, we will use latent class analyses [20-23] to predict a preterm birth when neither the GA based on the date of the LMP nor GA based on the ultrasound are treated as a reference standard. Finally, we will conduct classification and regression tree (CART) analyses to identify specific items from the T-SGAS and combine them with birth weight to achieve an improved prediction of a preterm birth, since birth weights are universally measured in health facilities soon after birth. The CART analyses will also provide an optimum protocol for implementation of the T-SGAS in community settings, since birth weights are now universally measured in the health facilities soon after birth. Improvement in discrimination and reclassification using the T-SGAS over the traditional method that uses birth weight and LMP will be quantified using the integrated discrimination improvement index and the net reclassification index, respectively [24,25].

## Results

The results of the T-SGAS development process will be as follows: (1) successful development of the Android app with iterative improvements based on feedback from end users; (2) successful training of ANMs, who are the main skilled birth attendants in Primary Health Centers and District and Regional level hospitals in India; and (3) successful implementation of the quality control procedures. Enrollment began on July 27, 2015 and ended on March 28, 2016. Results are anticipated in June 2019.

## Discussion

### Principal Findings

The T-SGAS and its evaluation by the research protocol has the potential to provide a new way for neonates born at first level care facilities and of unknown GA to be promptly referred to second or tertiary level care facilities, if the baby is preterm. Access to optimal care for preterm babies has the potential to improve outcomes in this at-risk population. An anticipated strength of the development of the app is the iterative development process based on user feedback. An anticipated strength of the evaluation is the large sample size of neonates that will be studied and the future analysis of the ability of ANMs to accurately assess GA using the app. If effective, the T-SGAS has the potential for rapid scale-up and the app could easily be modified to efficiently transfer medical data to referral facilities.

An important anticipated limitation of this study is that most fetal ultrasounds in India are obtained in the third trimester, instead of the ideal timing in the first trimester, in part to reduce the risk of the abortion of female fetuses. Whenever a first trimester ultrasound is done, we will obtain that report for GA dating and plan to conduct a subgroup analysis on the subset of pregnancies for which a first trimester ultrasound is available, providing there are sufficient first trimester ultrasound studies. We also recognize that prospectively collected data on the LMP or recall of the LMP earlier in pregnancy, as well as a first trimester ultrasound, could all improve the accuracy of the GA assessment. Conversely, the inability to accurately recall the LMP can impact study enrollment and is considered to be a further limitation of this study. However, the accuracy of all ultrasounds will likely be improved by requiring the GA by ultrasound being within 1 week of the mothers’ assessment of GA, based on her LMP.

### Conclusions

This paper describes the development of the T-SGAS and the research protocol to evaluate its use by ANMs in 3 second (District and Regional) level hospitals in Nagpur, India. Key outcomes will include whether the ANMs can correctly identify preterm babies using the app, compared to GA assessments by ultrasound obtained during pregnancy. This approach has the potential to improve outcomes in preterm infants born in first (Primary Care Centers) level facilities where their GA at birth may not be known if ANMs can accurately use the T-SGAS to assess GA and promptly refer those babies that are born prematurely.

## Abbreviations

ANM: auxiliary nurse midwife
CART: classification and regression tree
DWS: Dubowitz Score
FTP: File Transfer Protocol
GA: gestational age
ID: identification number
IRB: Institutional Review Board
LMP: last menstrual period
mHealth: mobile health
NBS: New Ballard Score
NICHD: National Institute of Child Health and Human Development
SGAS: simplified gestational age scoring system
T-SGAS: tablet app for the simplified gestational age scoring system
